# The impact of vascular volume fraction and compressibility of the interstitial matrix on vascularised poroelastic tissues

**DOI:** 10.1007/s10237-023-01742-1

**Published:** 2023-08-17

**Authors:** Pietro Mascheroni, Raimondo Penta, José Merodio

**Affiliations:** 1 Laboratoire Interdisciplinaire de Physique, Université Grenoble Alpes, 140, Rue de la Physique, 38402 Saint Martin d’Héres, France; 2 School of Mathematics and Statistics, University of Glasgow, University Place, Glasgow, G12 8QQ UK; 3 Departamento de Matemática Aplicada a las TIC ETS de Ingeniería de Sistemas Informáticos, Universidad Politécnica de Madrid, 28031 Madrid, Spain

**Keywords:** Poroelasticity, Asymptotic homogenization, Multiscale modelling, Vascular tumours

## Abstract

In this work we address the role of the microstructural properties of a *vascularised* poroelastic material, characterised by the coupling between a poroelastic matrix and a viscous fluid vessels network, on its overall response in terms of pressures, velocities and stress maps. We embrace the recently developed model (Penta and Merodio in Meccanica 52(14):3321–3343, 2017) as a theoretical starting point and present the results obtained by solving the full interplay between the *microscale*, represented by the intervessels’ distance, and the *macroscale*, representing the size of the overall tissue. We encode the influence of the vessels’ density and the poroelastic matrix compressibility in the poroelastic coefficients of the model, which are obtained by solving appropriate periodic cell problem at the microscale. The double-poroelastic model (Penta and Merodio 2017) is then solved at the macroscale in the context of vascular tumours, for different values of vessels’ walls permeability. The results clearly indicate that improving the compressibility of the matrix and decreasing the vessels’ density enhances the transvascular pressure difference and hence transport of fluid and drug within a tumour mass after a transient time. Our results suggest to combine vessel and interstitial normalization in tumours to allow for better drug delivery into the lesions.

**Supplementary Information:**

The online version contains supplementary material available at 10.1007/s10237-023-01742-1.

## Introduction

The mechanical response of solid, deformable, media interacting with fluid flowing within their pores is typically dealt with by means of the theory of poroelasticity (Biot 1955, 1956a, b, 1962), and its numerous extensions developed in the past few decades. Such poroelastic systems are of paramount importance in the context of biomechanical problems, as they provide a sound basis to investigate the mechanical response of tissues (Byrne and Preziosi 2003), cells (Moeendarbary et al. 2013) and extracellular matrices (Han et al. 2011).

While meaningful parameters related to stiffness, solid/fluid volume changes, porosity and overall fluid hydraulic conductivity, can be sometimes estimated on the basis of experimental measurements (see, e.g. Jain and Baxter 1988; Netti et al. 1995; Jain et al. 2007 in the context of solid tumours), these are usually related to fields such as velocities, pressure and displacements evaluated at a *macroscale* level and performed on the basis of simplified models which cannot always accurately consider the actual role of the underlying pore structure at the *microscale*.

As such, it is desirable to provide a link between the pore and overall scales characterising the material in order to ensure that experimental results concerning real-world physical systems can be interpreted in the light of the properties and behaviour of the microstructure. This way, it becomes possible to enhance the reliability of routinely adopted experimental setups, as done in Dehghani et al. (2020), where the authors show that parameters measurements performed over a not sufficiently long period of time can be highly inaccurate in terms of matrix compressibility. In addition, multiscale modelling strategies support the formulation of predictions which can inform the optimal microstructural arrangements to aim at target mechanical properties, as in Penta et al. (2016) and Miller and Penta (2023).

In fact, while the theory of poroelasticity was first derived on the basis of reasonable physical principles, it was subsequently formalised and rigorously derived by means of various averaging upscaling techniques, including Representative Elementary Volume methods, Mixture Theory and Asymptotic Homogenisation, see, e.g. Cheng and Cheng (2016), Rajagopal (2007), Burridge and Keller (1981) and Penta et al. (2020) and references therein. This latter approach is particularly suitable to obtain closed formulas relating the poroelastic coefficients with auxiliary quantities which can be obtained via solving differential problems on a limited portion of the pore-scale domain when local periodicity applies.

In Dehghani et al. (2018), the authors perform a systematic study by computing the poroelastic coefficients arising via the asymptotic homogenisation technique numerically and primarily focussing on the interplay between compressibility and porosity and its relevance in the context of biomaterials and (tumour) tissue mechanics.

However, real-world systems such as biological tissues are usually hierarchical in nature, which means that they can exhibit different geometrical and functional properties across multiple scales, and that their overall behaviour depends on the complex interactions that happen between these various levels of organisation.

There has been a substantial focus on hierarchical modelling in poroelasticity in the past decades (Pena et al. 1998; Cowin et al. 2009), with an emphasis on systems where the pore-scale structure can only be clearly resolved at the finest level of organisation. In fact, the authors in Rohan et al. (2016) addressed multiscale modelling of the interaction between a poroelastic compartment and a Newtonian fluid by means of the asymptotic homogenisation technique. In particular, they focus on two subsequent applications of the two-scale asymptotic homogenisation technique, rather than employing a three-scale multiscale approach from the commencement, as done for instance in Ramírez-Torres et al. (2018, 2019a, 2019b) in the context of elastic composites and bone modelling. The work Zampogna et al. (2019) also concerns a model derived by analogous assumptions but with an emphasis of the numerical computations of the effective properties and their influence on wave propagation. In 2021, Miller and Penta (2021a) derived an effective model for the interactions between two poroelastic phases, thus generalising the previous works Royer et al. (2019) and Chen et al. (2020) which addressed the interaction between a solid and a poroelastic compartment.

In Penta and Merodio (2017) the authors derived a double-poroelastic model characterised by mass exchange between the interstitial compartments and the vessels via upscaling a fluid structure interaction problem between a poroelastic matrix and a Newtonian fluid flowing in a network of interconnected vessels. By considering the walls of the vessels as a semi-permeable membrane, they generalised the results in Shipley and Chapman (2010) to deduce a new model which is relevant for deformable porous tissues percolated by a network of vessels such as vascularised tumours. The model in Penta and Merodio (2017) provides a link between the microstructure, characterised by the intercapillary distance (the *microscale*), and the *macroscale*, where the difference between the vessels and the poroelastic matrix can no longer be fully appreciated.

Here we present a set of results concerning the numerical solution of the model presented in Penta and Merodio (2017). Our simulations describe the mechanical response of the biological system at the tissue scale, informed by microstructural changes in terms of vascular volume fraction and compressibility of the interstitial poroelastic matrix. This is done by fully embracing the link between the micro- and macroscales in that the microscale cell problems derived in Penta and Merodio (2017) are solved for relevant ranges of both the vessels’ volume fraction and the Poisson ratio of the matrix by following the approach illustrated in Dehghani et al. (2018). The macroscale effective parameters in terms of stiffness, hydraulic conductivities and Biot’s moduli and coefficients are then computed on the basis of the microscale results. The macroscale problem is then solved for an in silico tissue specimen to investigate the time-dependent behaviour of pressures, relative velocities and stresses for various microstructural configurations. The results are finally illustrated in the context of tumour modelling in particular by investigating the interplay between vascular density, matrix compressibility and vessels’ permeability and its implications on the design of efficient anti-cancer therapies. In Sect. 2 we summarise the multiscale double-poroelastic model derived in Penta and Merodio (2017) and specialise the functional form of the coefficients for a fully interconnected vessels’ network which is invariant under permutations of the three coordinate axes. In Sect. 3 we present the results of our analysis at both the micro- and the macroscale. We conclude by summarizing our findings and illustrating further development of our work in Sect. 4.

## Methods

**Fig. 1:**
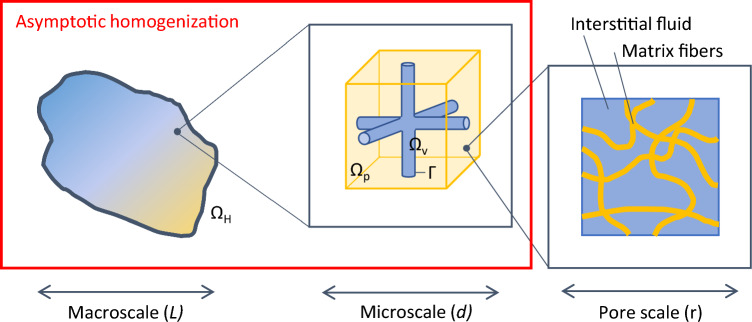
Macroscopic scale ($\Omega _\textrm{H}$) and zoom over the microscale, where the vascular ($\Omega _\textrm{v}$) and poroelastic ($\Omega _\textrm{p}$) domains are separated by the interface $\Gamma$. The zoomed region shows an example of the periodic cells used in the cell problems. At the pore scale, the solid matrix is constituted by a dense fibre network and is filled by interstitial fluid. We assume all the scales to be well-separated, i.e. $L \gg d \gg r$, and apply asymptotic homogenisation from the micro- to the macro-scale

In Penta and Merodio (2017) the authors present a mathematical model for the behaviour of vascularised poroelastic materials. This class of deformable solids is characterised by the presence of a porous skeleton, filled with fluid, that embeds a network of channels (e.g. vessels in biological applications), see Fig. 1.

The model is derived from the fluid–structure interaction between an isotropic and homogeneous Biot’s poroelastic compartment (Biot 1955, 1956a, b, 1962) and an incompressible Newtonian fluid, flowing in the network of vessels. The two compartments are coupled by interface conditions that take into account global conservation of mass and momentum, slip of the fluid over a porous surface and fluid transport across the interface between the two compartments (Penta and Ambrosi 2015), i.e. the blood vessels and the tumour interstitial space, in our setting. Then, the asymptotic homogenisation technique is exploited to derive the effective governing equations for the material at the macroscopic scale. In particular, it is assumed that the microscale characteristic length *d*, related to the distance between adjacent vessels, is well separated from the tissue macroscale characteristic length *L*, over which only global variations of the fields are relevant. This allows to decouple the two scales by introducing a small-scale separation parameter $\varepsilon$, such that

1 $$\begin{aligned} \varepsilon = \frac{d}{L} \ll 1, \quad {\varvec{y}}=\frac{{\varvec{x}}}{\varepsilon }. \end{aligned}$$

In (1), ${\varvec{x}}$ and ${\varvec{y}}$ are the two formally independent macroscale and microscale spatial variables, respectively (Sanchez-Palencia 1983). The problem can then be upscaled by relying on the typical steps involved in the asymptotic homogenisation technique, namely: the relevant fields are represented in power series of $\varepsilon$, and they are assumed to be functions of both ${\varvec{x}}$ and ${\varvec{y}}$. Then, the system of equations is non-dimensionalised according to suitable characteristic quantities in terms of length scales and velocity fields. After that, by equating the coefficients of the same power of $\varepsilon$ for $\varepsilon =0,1,\dots$ in the resulting system of PDEs one obtains a number of differential conditions. Finally, these conditions are used to obtain a closed differential problem for the leading (i.e. zeroth) order components of the fields. Notably, the coefficients that appear in the final equations encode the information on the microstructure of the material, as they are obtained by solving the microscale differential problems originating from the upscaling process (Cheng and Cheng 2016; Rajagopal 2007; Burridge and Keller 1981; Penta et al. 2020). The final set of PDEs describes the effective behaviour of the homogenised material, in terms of elastic displacements, pore and vascular pressures, and average fluid velocities. To this regard, the governing system of equations in the macroscale domain $\Omega _\textrm{H}\in \mathbb {R}^3$ for the leading-order contribution to the elastic displacement ${\varvec{u}}^{(0)}$, vascular and pore pressures $p^{(0)}_\textrm{v}$, $p^{(0)}_\textrm{p}$ reads

2 $$\begin{aligned}&\nabla _x \cdot \textsf{T}_\textrm{vp} = 0, \end{aligned}$$

3 $$\begin{aligned}&\dfrac{\dot{p}^{(0)}_\textrm{v}}{M_\textrm{vp}} = -\nabla _x \cdot \langle {\varvec{w}}^{(0)}_v\rangle _\textrm{v} - \textsf{A}_\textrm{v} : \nabla _x \dot{{\varvec{u}}}^{(0)} + \dfrac{\dot{p}^{(0)}_\textrm{p}}{M_\textrm{vp}} - \dfrac{|\Gamma |\bar{L}_\textrm{p}}{|\Omega |}\left( p^{(0)}_\textrm{v}-p^{(0)}_\textrm{p}\right) , \end{aligned}$$

4 $$\begin{aligned}&\dfrac{\dot{p}^{(0)}_\textrm{p}}{M_\textrm{vp}} = -\nabla _x \cdot \langle {\varvec{w}}^{(0)}_\textrm{p}\rangle _\textrm{p} - \textsf{A}_\textrm{p} : \nabla _x \dot{{\varvec{u}}}^{(0)} + \dfrac{\dot{p}^{(0)}_n}{M_\textrm{vp}} + \dfrac{|\Gamma |\bar{L}_\textrm{p}}{|\Omega |}\left( p^{(0)}_\textrm{v}-p^{(0)}_\textrm{p}\right) , \end{aligned}$$

where $\dot{p}^{(0)}_\textrm{v}$, $\dot{p}^{(0)}_\textrm{p}$ and $\dot{{\varvec{u}}}^{(0)}$ denote the time derivatives of the pressure fields and solid displacement, $\langle {\varvec{w}}^{(0)}_v\rangle _\textrm{v}$ and $\langle {\varvec{w}}^{(0)}_p\rangle _\textrm{p}$ are the average fluid velocities in the vascular and poroelastic compartments, and $\textsf{T}_\textrm{vp}$ is the total stress in the tissue. Then, $\Gamma$ and $\Omega$ are the exchange surface and volume of the unit microscopic cell, whereas $\bar{L}_\textrm{p}$ is the non-dimensional vascular permeability. Finally, the variables $M_\textrm{vp}$, $\textsf{A}_\textrm{v}$ and $\textsf{A}_\textrm{p}$ are effective Biot’s-type poroelastic coefficients whose relationship with the microstructure will be discussed in the remainder of this section.

The constitutive relation for the total stress $\textsf{T}_\textrm{vp}$ in the homogenised material is given by

5 $$\begin{aligned} \textsf{T}_\textrm{vp} = \tilde{\mathbb {C}}:\nabla _x {\varvec{u}}^{(0)} - \textsf{A}_\textrm{v} p^{(0)}_\textrm{v} - \textsf{A}_\textrm{p} p^{(0)}_\textrm{p}, \end{aligned}$$

where $\tilde{\mathbb {C}}$ is the effective elasticity tensor. Then, the average fluid velocity in the vascular and poroelastic compartments is obtained as

6 $$\begin{aligned} \langle {\varvec{w}}^{(0)}_\textrm{v}\rangle _\textrm{v}&= - \textsf{K} \nabla _x p^{(0)}_\textrm{v}, \end{aligned}$$

7 $$\begin{aligned} \langle {\varvec{w}}^{(0)}_\textrm{p}\rangle _\textrm{p}&= - \bar{k}\textsf{G} \nabla _x p^{(0)}_\textrm{p}, \end{aligned}$$

where $\textsf{K}$ and $\bar{k}\textsf{G}$ are the effective hydraulic conductivity tensors of the vascular and poroelastic compartments, while $\bar{k}$ is the non-dimensional hydraulic conductivity of the isotropic poroelastic matrix.

Equations (2)–(7) describe a double-poroelastic homogenised material, in which (2) represents the balance of linear momentum, while (3) and (4) account for the fluid exchange across the vascular and poroelastic compartments. In the following, we briefly describe the significance of the homogenised coefficients and the derivation process.

The upscaling process naturally leads to the constitutive relation in Eq. (5) for the total stress and to Darcy-like laws (6) and (7) for the vascular and pore average velocities. The non-dimensional numbers $\bar{L}_\textrm{p}$ and $\bar{k}$ are defined by

8 $$\begin{aligned} \bar{L}_\textrm{p} = \frac{L_\textrm{p}\mu L^2}{d^3},\quad \bar{k} = \frac{k\mu }{d^2}, \end{aligned}$$

where $L_\textrm{p}$ and *k* are the physiological vascular hydraulic permeability and poroelastic hydraulic conductivity, respectively, and $\mu$ is the fluid viscosity. Both $\bar{L}_\textrm{p}$ and $\bar{k}$ play a key role in the biomechanical response of the tissue. On the one hand, higher values of $\bar{L}_\textrm{p}$ represent more permeable vessels—as in the case of tumours, where abnormal angiogenesis leads to leaky vasculature (Maeda 2015). On the other hand, the value of $\bar{k}$ controls fluid flow in the interstitial space and is related to the chemical and geometrical properties of this compartment (Jain 1990). The behaviour of the homogenised material is modulated by the effective elasticity tensor $\tilde{\mathbb {C}}$, the vascular and poroelastic Biot’s coefficients $\textsf{A}_\textrm{v}$ and $\textsf{A}_\textrm{p}$, the geometric Biot’s modulus $M_\textrm{vp}$, and the vascular and poroelastic hydraulic conductivities $\textsf{K}$ and $\textsf{G}$. All these quantities can be expressed in terms of suitable averages over the microscale domain, spanned by the spatial coordinate ${\varvec{y}}$. In practice, some regularity assumptions are enforced in order to compute the coefficients with a low computational cost. Following the traditional approach in asymptotic homogenisation for biological tissues (Shipley and Chapman 2010; Penta and Ambrosi 2015), we assume microscale periodicity (that is, with respect to the variable ${\varvec{y}}$) in the calculations. With this simplification, the coefficients introduced above can be calculated through integral averages performed on a single periodic cell. These are defined through the cell average operator

9 $$\begin{aligned} \langle \bullet \rangle _\textrm{j} = \frac{1}{|\Omega |}\int _{\Omega _\textrm{j}} \bullet \, \hbox {d}{\varvec{y}},\quad \textrm{j}=\textrm{v},\textrm{p}, \end{aligned}$$

where $\Omega$ is the periodic cell domain, with corresponding vascular and poroelastic subdomains $\Omega _\textrm{v}$ and $\Omega _\textrm{p}$, respectively. The scalars $|\Omega _\textrm{v}|$ and $|\Omega _\textrm{p}|$ are the vascular and poroelastic volumes in the unit cell ($|\Omega |=|\Omega _\textrm{v}|+|\Omega _\textrm{p}|$). We define the vascular and poroelastic volume ratios as

10 $$\begin{aligned} \phi _\textrm{v} = \frac{|\Omega _\textrm{v}|}{|\Omega |},\quad \phi _\textrm{p} = \frac{|\Omega _\textrm{p}|}{|\Omega |}, \end{aligned}$$

whereas the cell exchange surface $\Gamma$ is defined by the surface integral

11 $$\begin{aligned} |\Gamma |= \int _\Gamma \hbox {d}S_y, \end{aligned}$$

in which $\Gamma$ denotes the interface between the vascular and poroelastic compartments, i.e. $\Gamma =\partial \Gamma _\textrm{v} \cap \Omega _\textrm{p}$. Higher values of $|\Gamma |$ translate into an increased surface area that is available for solute and fluid exchange between vessels and interstitial space.

The macroscale coefficients in terms of averages of the microscale auxiliary variables read

12 $$\begin{aligned} \begin{aligned} \tilde{\mathbb {C}}&=\langle \mathbb {C}\mathbb {M} + \mathbb {C}\rangle _\textrm{p},\quad \textsf{A}_\textrm{v} = \phi _\textrm{v}\textsf{I} - \textrm{Tr}\langle \mathbb {M}\rangle _\textrm{p}, \quad \textsf{A}_\textrm{p} = \phi _\textrm{p}\textsf{I} + \textrm{Tr}\langle \mathbb {M}\rangle _\textrm{p},\\&M_\textrm{vp} = -\frac{1}{\textrm{Tr}\langle \textsf{Q} \rangle _\textrm{p}}, \quad \textsf{K}=\langle \textsf{W}\rangle _\textsf{v},\quad \textsf{G}=\phi _\textsf{p}\textsf{I} - \langle \textsf{P} \rangle _\textrm{p}. \end{aligned} \end{aligned}$$

The fourth-rank tensor $\mathbb {M}$ and the second-rank tensors $\textsf{Q}$, $\textsf{W}$ and $\textsf{P}$ that appear in (12) are computed by solving the microscale cell problems discussed in the next section. As such, they directly account for the influence of the unit cell microstructure on the tissue-level mechanical properties. In the next section we will show the necessary steps involved in their derivation.

### Cell problems

The analysis is performed in non-dimensional form, so that the cell problems for the auxiliary variables $\mathbb {M}$, $\textsf{Q}$, $\textsf{W}$ and $\textsf{P}$ are solved in a unit cubic cell $\Omega$. The latter is composed of the vascular domain $\Omega _\textrm{v}$, given by a cross-shaped cylindrical structure accounting for a fully connected porous medium, and the poroelastic domain $\Omega _\textrm{p}=\Omega /\Omega _\textrm{v}$. This geometry is the simplest configuration describing a fully three-dimensional flow in a saturated porous material. It also allows to keep to a minimum the number of macroscale parameters (see the schematics in Fig. 1).

#### Hydraulic conductivity tensors

The second-rank tensor $\textsf{W}$ involved in the calculation of the effective vascular hydraulic conductivity tensor $\textsf{K}$ (see Eq. (12)) is determined by solving the following auxiliary Stokes’-type cell problem on the microscale:

13 $$\begin{aligned}&\nabla _y {\varvec{P}}_\textrm{v} = \nabla _y^2 \textsf{W}^\textrm{T} + \textsf{I} \text{ in } \Omega _\textsf{v}, \end{aligned}$$

14 $$\begin{aligned}&\nabla _y \cdot \textsf{W}^\textrm{T} = 0 \text{ in } \Omega _\textsf{v},\end{aligned}$$

15 $$\begin{aligned}&\textsf{W}^\textrm{T} {\varvec{n}} = 0 \text{ on } \Gamma ,\end{aligned}$$

16 $$\begin{aligned}&\textsf{W}^\textrm{T}{\varvec{t}}_i = - \alpha ^{-1}\sqrt{\bar{k}}\left[ \left( \nabla _y \textsf{W}^\textrm{T} + (\textsf{W}^\textrm{T})^\textrm{T}\right) {\varvec{n}}\right] {\varvec{t}}_i \text{ on } \Gamma , \end{aligned}$$

where ${\varvec{n}}$ and ${\varvec{t}}_i$ are the unit vectors normal and tangential to the interface $\Gamma$, respectively, $\textsf{I}$ is the identity tensor, and ${\varvec{P}}_\textrm{v}$ is an auxiliary vector. The parameter $\alpha$ is the non-dimensional Beavers and Joseph coefficient (Penta and Merodio 2017), accounting for the boundary effects arising at the interface between the vessels and the (porous) interstitial space.

The cell problem for the second-rank tensor $\textsf{P}$, which appears in the definition of the effective interstitial hydraulic conductivity tensor $\textsf{G}$ defined in (12), is given by:

17 $$\begin{aligned}&\nabla _y^2 {\varvec{P}}_\textrm{p} = 0 \text{ in } \Omega _\textrm{p}, \end{aligned}$$

18 $$\begin{aligned}&(\textsf{I}-\nabla _y {\varvec{P}}_\textrm{p}){\varvec{n}} = 0 \text{ in } \Gamma , \end{aligned}$$

where the auxiliary vector ${\varvec{P}}_\textrm{p}$ is such that $\textsf{P}=(\nabla _y{\varvec{P}}_\textrm{p})^\textrm{T}$, cf. (12). Equations (13)–(18) are equipped with ${\varvec{y}}$-periodic conditions on the remaining part of the unit cell boundary and are supplemented by the uniqueness conditions

19 $$\begin{aligned} \langle {\varvec{P}}_\textrm{v} \rangle _\textrm{v} = 0,\quad \langle {\varvec{P}}_\textrm{p} \rangle _\textrm{p} = 0. \end{aligned}$$

We refer the interested readers to the discussion in Penta and Ambrosi (2015) and Dehghani et al. (2018) for additional details concerning the numerical implementation of the above cell problems.

#### Effective elasticity tensor

The fourth-rank tensor $\tilde{\mathbb {C}}$ describes the effective drained stiffness tensor for the homogenised material. To give an intuitive description of its biological significance, this tensor accounts for the contribution of the interstitial space to the total mechanical stress in the tissue. According to Eq. (12), $\tilde{\mathbb {C}}$ depends on the stiffness tensor of the poroelastic compartment and on the auxiliary tensor $\mathbb {M}$ defined as

20 $$\begin{aligned} \mathbb {M} = (\nabla _y \mathcal {A})_s, \end{aligned}$$

where $(\bullet )_s$ represents the symmetric part of the argument, and the third-rank tensor $\mathcal {A}$ is obtained from the following cell problem on the poroelastic compartment:

21 $$\begin{aligned}&\nabla _y \cdot (\mathbb {C}(\nabla _y \mathcal {A})_s) = 0 \text{ in } \Omega _\textrm{p}, \end{aligned}$$

22 $$\begin{aligned}&(\mathbb {C}(\nabla _y \mathcal {A})_s){\varvec{n}}+\mathbb {C}{\varvec{n}}=0 \text{ on } \Gamma ,\end{aligned}$$

23 $$\begin{aligned}&\langle \mathcal {A} \rangle _\textrm{p} = 0, \end{aligned}$$

in which the system is also equipped with periodic conditions on $\partial \Omega _\textrm{p}/\Gamma$. As thoroughly exposed in Dehghani et al. (2018), the problem in Eqs. (21)–(23) is formally equivalent to solve six elastic-type cell problems equipped with inhomogeneous Neumann interface conditions.

For the sake of simplicity, we limit the discussion to standard poroelasticity, in which the drained stiffness tensor $\mathbb {C}$ is the one of linear isotropic materials. Therefore, the latter is completely determined by assigning the (drained) Young modulus *E* and (drained) Poisson’s ratio $\nu$. Due to the assumption of isotropy, the invariance properties of the geometry dictate that both $\mathbb {M}$ and $\tilde{\mathbb {C}}$ possess cubic symmetry. In the following, we will describe the mechanical properties of the tissue in terms of the effective Young modulus $E_\textrm{ef}$, Poisson’s ratio $\nu _\textrm{ef}$ and shear modulus $\mu _\textrm{ef}$. These quantities are defined by the relations (Dehghani et al. 2018)

24 $$\begin{aligned} E_\textrm{ef}&= \dfrac{\tilde{C}_{11}(\tilde{C}_{11}+\tilde{C}_{12})-2\tilde{C}_{12}^2}{\tilde{C}_{11}+\tilde{C}_{12}}, \end{aligned}$$

25 $$\begin{aligned} \nu _\textrm{ef}&= \dfrac{\tilde{C}_{12}}{\tilde{C}_{11}+\tilde{C}_{12}},\end{aligned}$$

26 $$\begin{aligned} \mu _\textrm{ef}&= \tilde{C}_{44}. \end{aligned}$$

#### Vascular and poroelastic Biot’s coefficients

As per Eq. (12), the expression for the vascular and poroelastic Biot’s coefficients $A_\textrm{v}$ and $A_\textrm{p}$ requires the calculation of the trace of the auxiliary tensor $\mathbb {M}$. Thanks to the cubic symmetry, the latter can be calculated as

27 $$\begin{aligned} \textrm{Tr}\mathbb {M} = (M_{1111}+2M_{1122})\textsf{I}, \end{aligned}$$

so that both Biot’s effective coefficients assume a diagonal form, see Eq. (12).

#### Geometric Biot’s modulus

Finally, the geometric Biot’s modulus defined in Eq. (12) depends on the auxiliary tensor $\textsf{Q}$, which is given by

28 $$\begin{aligned} \textsf{Q} = \nabla _y {\varvec{a}}, \end{aligned}$$

in which ${\varvec{a}}$ is the solution of the periodic cell problem

29 $$\begin{aligned} \nabla _y\cdot (\tilde{\mathbb {C}}\nabla _y {\varvec{a}})&= 0 \text{ in } \Omega _\textrm{p}, \end{aligned}$$

30 $$\begin{aligned} (\tilde{\mathbb {C}}\nabla _y {\varvec{a}} + \textsf{I}){\varvec{n}}&= 0 \text{ on } \Gamma . \end{aligned}$$

As previously, the latter cell problem on the poroelastic compartment is formally equivalent to a linear elastic problem equipped with inhomogeneous Neumann interface conditions on $\Gamma$ and periodic conditions on $\partial \Omega _\textrm{p}/\Gamma$. For the solution of Eqs. (29) and (30) to be unique, we also require that

31 $$\begin{aligned} \langle {\varvec{a}} \rangle _\textrm{p} = 0. \end{aligned}$$

For the sake of brevity and clarity of presentation, we condensed the crucial derivation steps in a few pages. A more thorough traction of these aspects is available to the interested reader in Penta and Merodio (2017). In the following section we will present the numerical solutions of the aforementioned cell problems, as well as the solution of the macroscale problem in Eqs. (2)–(4) for a simple setting. The numerical solutions are obtained by coding the PDEs in the commercial software COMSOL Multiphysics^®^ (2021). We implemented the microscale cell problems in the solid mechanics module using quadratic serendipity finite elements. For the macroscale problem, we coupled the solid mechanics module to two ’Coefficient form PDE’ modules. Equations were discretized using quadratic Lagrange finite elements. Time discretization was carried out via an implicit backward differentiation formula. In both the microscale cell problems and macroscale problems the discretized equations were solved as a fully coupled system, making use of a standard Newton solver.

#### Comparison with existing modelling frameworks

The system of PDEs in (2)–(4) can be seen as a generalization of previous multiscale models that were derived in the context of porous media. On the one hand, when the porous solid matrix is assumed to be rigid, the leading-order contribution to the solid displacement ${\varvec{u}}^{(0)}$ can be set to zero. In this latter case, the model can be written in terms of the two leading-order vascular and interstitial fluid pressures only, i.e. $p_\textrm{v}^{(0)}$ and $p_\textrm{p}^{(0)}$, respectively. As the geometric Biot’s modulus $M_\textrm{vp}$ tends to infinity for a rigid matrix, the simplified macroscale model reads:

32 $$\begin{aligned}&\nabla _x \cdot \left( \tilde{\textsf{K}} \nabla _x p^{(0)}_\textrm{v} \right) = \dfrac{|\Gamma |\bar{L}_\textrm{p}}{|\Omega |}\left( p^{(0)}_\textrm{v}-p^{(0)}_\textrm{p}\right) , \end{aligned}$$

33 $$\begin{aligned}&\nabla _x \cdot \left( \tilde{\textsf{G}} \nabla _x p^{(0)}_\textrm{p} \right) = \dfrac{|\Gamma |\bar{L}_\textrm{p}}{|\Omega |}\left( p^{(0)}_\textrm{p}-p^{(0)}_\textrm{v}\right) , \end{aligned}$$

where the effective conductivities are rescaled to account for the intrinsic average operator. Therefore, simplified model (32–33) coincides, up to a slightly different notation, with the double Darcy macroscale problem derived in Shipley and Chapman (2010) and Penta et al. (2015) which describes mass exchange in vascularised rigid tumours.

On the other hand, the model in (2)–(4) can also be regarded as a generalization of the standard Biot’s equations for poroelasticity with coefficients derived from the microstructure, see Burridge and Keller (1981). Assuming that there is no mass exchange between compartments and no interstitial fluid within the matrix, it is possible to rewrite the equations in terms of the fluid pressure $p_\textrm{v}^{(0)}$ and the solid displacement ${\varvec{u}}^{(0)}$ only. This is because in this case $p_p=p_p^{(0)}=\phi _p=\bar{L}_p=0$, and since $\phi _p=0$ also implies that $\textsf{A}_p=\textsf{0}$, the system of the equations reduces to

34 $$\begin{aligned}&\nabla _x \cdot \textsf{T}_\textrm{vp} = 0, \end{aligned}$$

35 $$\begin{aligned}&\dfrac{\dot{p}^{(0)}_\textrm{v}}{M_\textrm{vp}} = -\nabla _x \cdot \langle {\varvec{w}}^{(0)}_v\rangle _\textrm{v} - \textsf{A}_\textrm{v} : \nabla _x \dot{{\varvec{u}}}^{(0)}, \end{aligned}$$

where the average vessels’ velocity $\langle {{\varvec{w}}}^{(0)}_v\rangle$ is driven by the vascular pressure gradient according to Darcy’s law (6), while the constitutive relationship for $\textsf{T}_{vp}$ simplifies to

36 $$\begin{aligned} \textsf{T}_\textrm{vp} = \tilde{\mathbb {C}}:\nabla _x {\varvec{u}}^{(0)} - \textsf{A}_\textrm{v} p^{(0)}_\textrm{v}. \end{aligned}$$

The auxiliary tensors $\mathbb {M}$ and $\textsf{Q}$ which appear in the definition of the coefficients [cf. Eq. (12)] are then to be solved by computing problems analogous to those illustrated in Dehghani et al. (2018). In this case, the system of PDEs (34–35) formally reads as a standard poroelastic model where the vascular compartment plays the role of the interstitial fluid compartment percolating through an otherwise solid elastic matrix.

## Results

This section is divided in two parts. First, we present the results of the model in terms of effective tensors, which are calculated from the cell problems in the Sect. 2. We remark that the effective tensors encode the contribution of the microscale structure—interstitial space and blood vessels—to the poromechanical response of the tissue. As such, they convey a profound biological significance in the modelling framework. Then, we report on the solution of the macroscale equations in Eqs. (2)–(4) for a simple macroscale problem of stress relaxation. We show how a tissue specimen relaxes subjected to a boundary load under a change of microstructural properties and independent biological parameters. The analysis is performed in non-dimensional form, and the dimensional counterpart of each parameter can be calculated as per the non-dimensionalisation discussed in the Sect. 2.

Further considerations concerning the rationale behind our analysis follow below. Given the multiscale nature of the modelling approach, the vascular permeability ($L_{\textrm{p}}$, or $\bar{L}_{\textrm{p}}$ in non-dimensional form) is the most relevant parameter which plays a crucial role on the tumour mechanics and, at the same time, is not to be computed based on a micromechanical approach in the present formulation. The remaining effective macroscale parameters (stiffnesses, Biot’s moduli, hydraulic conductivities, surface-to-volume ratios) are to be computed based on the microstructure (i.e. its geometry, matrix and vessels volume fractions, and microscale properties such as stiffness and hydraulic conductivities), cf. system of PDEs (2–4), as supplemented by Eqs. (5), (6) and (7), as well as relationship (12) which illustrates the fact that all (but $L_p$) parameters are obtained by means of analytical formulae which require microscale computations of suitable auxiliary variable. As far as this particular work is concerned, at the microscale, we have therefore focussed on the compressibility of the matrix $\nu$ (and also on its stiffness, although our analysis with respect to the microscale Young modulus is aligned with previous results and variations of this latter produce fairly intuitive consequences, so that we relegated such results in Online Appendix A), and the vascular volume fraction $\phi _v$ and discussed their implications on the macroscale results. These latter parameters are indeed being varied according to suitable physiological ranges. Furthermore, at the macroscopic scale we have then also focussed on the vascular permeability. The importance of the letter parameter on the tissue biomechanics is clearly evident from previous investigations, and there is also a large experimental literature concerning the influence of vascular permeability in the context of drug delivery, as this serves as a validation benchmark for model results, see, e.g. Mascheroni and Penta (2017), Penta and Ambrosi (2015), Jain et al. (2007) and references therein.

### Solution of the cell problems

Equations (13)–(16), (17)–(18), (21)–(23), (29)–(30) for the cell problems are solved in their corresponding subsets of the cubic unit cell $\Omega$, i.e. the vascular domain $\Omega _\textrm{v}$ and the poroelastic domain $\Omega _\textrm{p}$. We explore the parameter space that is generated by varying the drained Young modulus *E*, Poisson ratio $\nu$ and vascular volume fraction $\phi _\textrm{v}$ within a feasible physiological range (Islam et al. 2020; Penta and Ambrosi 2015). In particular, we focus on the impact of microscale compressibility and vascular volume fraction on the tissue mechanical properties and poroelastic moduli. Therefore, we will present the results for a fixed value of the Young modulus *E*, leaving the thorough analysis in the Supplementary (see Fig. S1 and S2). In the following discussion, *E* is set to 27.5, corresponding to a dimensional value of 55kPa—in the physiological range of soft tissues (Islam et al. 2020). A reference interstitial pressure of 2 kPa is taken into account for the non-dimensionalisation (Jain and Baxter 1988). Concerning the vascular volume ratio, the parametric analysis is performed by varying the radius of three interconnected cylinders (see Fig. 1) to obtain $\phi _\textrm{v}$ in the range [0.01, 0.1] (see, e.g. Gullino and Grantham 1964; Meyer et al. 1993). With respect to the drained Poisson ratio $\nu$, we consider the range $\nu \in$[0.2, 0.4], exploring a wide physiological range (Islam et al. 2020).

#### Mechanical parameters

The calculation of the hydraulic conductivity tensors $\textsf{K}$ and $\textsf{G}$ of Eq. (12) is performed as thoroughly described in Penta and Ambrosi (2015) and Dehghani et al. (2018). These effective tensors are independent from the drained Poisson ratio of the unit cell, but show a strong dependency on the vascular volume ratio (see Fig. S3). In particular, there is a nonlinear drop of the conductivity in the vascular space $\textsf{K}$ for decreasing values of $\phi _\textrm{v}$, whereas the conductivity in the poroelastic compartment $\textsf{G}$ increases linearly for increasing values of the vascular volume ratio. The model is predicting that larger vascular fractions lead to an increased contribution to fluid transport from the vascular compartment and a decreased contribution from the interstitial space. As the two trends occur following different functional forms (see Fig. S3), non-trivial transport behaviour might emerge.

**Fig. 2:**
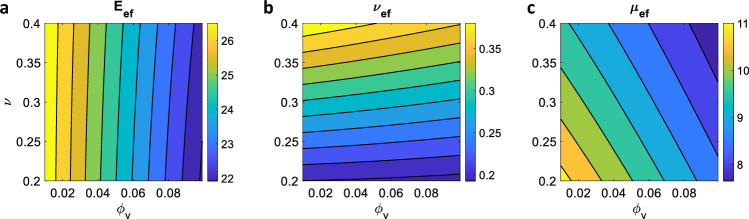
Effective Young modulus (**a**), Poisson ratio (**b**), and shear modulus (**c**) calculated from the cell problems at the microscale. In each plot, the quantity of interest is evaluated for different values of vascular fraction ($\phi _\textrm{v}$) and compressibility ($\nu$). For each case, we selected an intermediate value of the microscale Young modulus (i.e. $E=27.5$)

Figure 2 shows the variation of effective Young modulus ($E_\textrm{ef}$), Poisson ratio ($\nu _\textrm{ef}$) and shear modulus ($\mu _\textrm{ef}$) against vascular volume ratio and drained Poisson ratio. These effective quantities are defined in Eqs. (24)–(26) and depend both on the mechanical and geometric properties of the poroelastic compartment. Both Young and Poisson moduli decrease for increasing vascular volume rations (see Fig. 2a, b) as the homogenized material becomes more and more compliant towards higher vascularisations. Indeed, both $E_\textrm{ef}$ and $\nu _\textrm{ef}$ represent *drained* quantities in a poroelastic mindset, i.e. they quantify the compliance of the homogenised material when its pores are thought as empty. The effective Young modulus displays a less dramatic dependence on the drained Poisson modulus (*y* axis in Fig. 2a), differently from the effective Poisson ratio shown in Fig. 2b. Finally, the effective shear modulus in Fig. 2c varies both along the $\phi _\textrm{v}$ and $\nu$ coordinates. Homogenised materials with high vascular ratio and low compressibility display the lower values of $\mu _\textrm{ef}$, i.e. resist less to shear deformations.

#### Poroelastic moduli

**Fig. 3:**
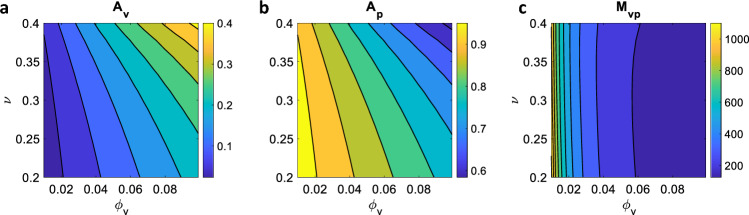
Effective vascular (**a**) and poroelastic (**b**) Biot coefficients, and geometric Biot modulus (**c**). In each plot, the quantity of interest is evaluated for different values of vascular fraction ($\phi _\textrm{v}$) and compressibility ($\nu$). For each case, we selected an intermediate value of the microscale Young modulus (i.e. $E=27.5$)

The vascular and poroelastic effective Biot’s coefficients ($A_\textrm{v}$, $A_\textrm{p}$, respectively) and the geometric Biot’s modulus $M_\textrm{vp}$ are displayed in Fig. 3 for different values of vascular volume ratio and drained Poisson modulus. These effective quantities, defined in Eq. (12), modulate the homogenised material response in terms of poromechanical effects. In particular, $A_\textrm{v}$ and $A_\textrm{p}$ weight the contribution of vascular and pore pressure, respectively, to the homogenised total stress, see Eq. (5). They are the counterpart of the Biot’s coefficient in standard poroelasticity (Biot 1955, 1956a, 1962) and are related to the compressibility of the material constituents. As such, $A_\textrm{v}$ reaches higher values for high vascularisations, when the (incompressible) fluid content fraction is higher (see Fig. 3a). The vascular Biot coefficient also increases for increasing values of the drained Poisson ratio, with slower variations at low $\phi _\textrm{v}$ and faster rates for higher vascular volume ratios.

From the definitions of $A_\textsf{v}$ and $A_\textrm{p}$ in Eq. (12) and the saturation constraint (i.e. $\phi _\textrm{v}\,+\,\phi _p=1$), we have that $A_\textrm{p}=1-A_\textrm{v}$, explaining the profile in Fig. 3b. In particular, for low values of $\phi _\textrm{v}$ we recover the behaviour in the ’pure’ poroelastic domain, i.e. $A_\textsf{p}\sim 1$. As the vascular ratio increases, the fluid component weights more in the homogenised response, and the proportion of total stress that is carried by the interstitial and vascular pressures changes.

Finally, Fig. 3c shows the variation of the geometric Biot’s modulus $M_\textrm{vp}$ against vascular volume fraction and drained Poisson ratio. Through Eqs. (3) and (4), this quantity relates the variation in fluid content in the homogenised material to the variation in pressure difference between the two compartments (Penta and Merodio 2017). From its definition in Eq. (12), it is related to the poroelastic elasticity tensor and the geometry of the microstructural problem. As the vascular volume decreases, $M_\textrm{vp}$ tends to higher values, with the limit $M_\textrm{vp}\rightarrow \infty$ for a material entirely constituted by the poroelastic compartment, in which we set the Biot modulus to infinity. There is a strong dependence of $M_\textrm{vp}$ on $\phi _\textrm{v}$, with the geometric Biot modulus decreasing rapidly at increasing values of $\phi _\textrm{v}$. On the other hand, $M_\textrm{vp}$ depends only weakly on the value of the drained Poisson ratio for the explored parameter range. Note that, in view of the results reported in Dehghani et al. (2018), we expect a non-trivial dependence of $M_\textrm{vp}$ on $\phi _\textrm{v}$ at vascular volumes larger than 0.1, with the presence of a minimum before the geometric Biot modulus increases again to infinity for $\phi _\textrm{v}\rightarrow 1$ (where the poroelastic compartment is not present anymore).

**Fig. 4:**
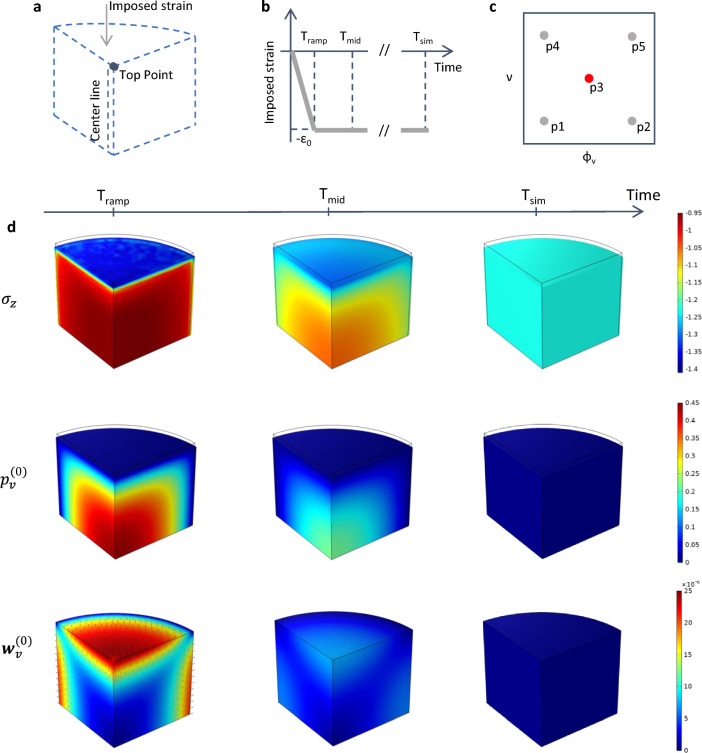
Schematic of the three-dimensional problem solved in the macroscale simulations (**a**). We impose a compressive strain on the top surface of the material, together with imposing zero fluid pressure for both the vascular and poroelastic compartments. On the symmetry faces we prescribe no normal displacements and no flux conditions. The lateral surface can deform freely, and we also set the fluid pressure to zero in both compartments. The time dependency of the imposed strain is shown in the inset in **b**. We highlight three time points, namely $T_\mathrm{{ramp}}$, $T_\mathrm{{mid}}$ and $T_\mathrm{{sim}}$, which represent the end of the loading ramp, an intermediate time position and the end of the simulation, respectively. **c** Schematics of the combinations of the microscale parameters $(\phi _\textrm{v},\,\nu )$ that have been investigated in the macroscale computations. The red dot (p3) refers to the quantities that are plotted in **d**. In particular, we show the temporal evolution of the vertical component of the solid stress $(\sigma _z)$, pressure $(p^{(0)}_f)$, and fluid velocity $({\varvec{w}}^{(0)}_f)$ in the vascular compartment

### Macroscale problem

In this section we discuss the solution of Eqs. (2)–(4) for a reference macroscale problem. In particular, we focus on a stress relaxation experiment, schematised in Fig. 4a. We simulate the compression of a cylindrical tissue specimen, which is squeezed between two parallel plates. The symmetries of the problem allow to simulate only one quarter of the specimen, as highlighted in the figure. The material is at rest at time zero, with both vascular ($p^{(0)}_\textrm{v}$) and pore pressures ($p^{(0)}_\textrm{p}$) set to zero, as for the solid displacements (${\varvec{u}}^{(0)}$). Regarding boundary conditions, we use symmetry conditions on the internal and bottom surfaces of the quarter of cylinder. The curved face at the exterior of the cylinder is traction-free, and we set both $p^{(0)}_\textrm{v}$ and $p^{(0)}_\textrm{p}$ to zero. We also set to zero the pressures at the top surface, and we impose there a vertical compressive strain which depends on time as depicted in Fig. 4b. We impose a negative strain that reaches its minimum value $\varepsilon _0$ at $t=T_\textrm{ramp}$. This strain is then kept over time, while the homogenised material reaches an equilibrium as in standard stress relaxation experiments (see e.g. Fung 2013). We analyse three temporal snapshots of the macroscale solution at $T_\textrm{ramp}$, $T_\textrm{mid}$ and $T_\textrm{sim}$, providing a series of snapshots over the dynamics of the stress relaxation process. In addition, we consider different positions in the parameter space of the microscale parameters $\phi _\textrm{v}$ and $\nu$, i.e. the vascular volume ratio and drained Poisson ratio (see Table 1 and Fig. 4c). This allows to appreciate the poroelastic effects that characterise the relaxation process depending on variation of physiologically relevant parameters.

**Table 1:** Microscale Poisson ratio and vascular volume ratio used in the macroscale simulations

Case	$\nu$	$\phi _\textrm{v}$
p1	0.22	0.018
p2	0.22	0.092
p3	0.31	0.051
p4	0.37	0.018
p5	0.37	0.092

Figure 4d shows the macroscale results for intermediate values of $\phi _\textrm{v}$ and $\nu$ (red point in Fig. 4c) over time. The first row in Fig. 4d shows the vertical component of the homogenised solid stress in the material (i.e. $\sigma _z=(\tilde{\mathbb {C}}:\nabla {\varvec{u}}^{(0)})_{zz}$) over the macroscale domain. As time increases, the tensional state evolves towards a homogeneous condition, with $\sigma _z$ increasing in the centre of the cylinder as the fluid escapes from the specimen. Basically, the early part of the load is carried by the fluid in the vessels and interstitial pores. As time progresses, the solid component of the tissue takes increasing portions of the load. Note that the adoption of a cubic cell microstructural geometry in the cell problems in the Sect. 2 produces a cubic anisotropy in the homogenised stiffness tensor $\tilde{\mathbb {C}}$, which affects the symmetry of the solution (see Penta and Gerisch 2015; Dehghani et al. 2020 for a detailed discussion of this effect). In the second row of Fig. 4d we show the evolution of the vascular pressure at the three time points that have been analysed. This quantity behaves in a specular way compared to the solid stress, reaching the highest value at the centre of the specimen rightly after the end of the loading phase. After that, it relaxes and equilibrates with the pressure outside of the material. Finally, the last row of Fig. 4d displays the temporal variation of fluid velocity in the vascular compartment, ${\varvec{w}}^{(0)}_\textrm{v}$. The colour map shows the modulus of ${\varvec{w}}^{(0)}_\textrm{v}$, whereas the red arrows denote the velocity vectors. The fluid velocity is obtained from the vascular pressure by Eq. (6), following a Darcy-like relation. Higher values of fluid velocity are obtained in the regions of faster variation of the vascular pressure, reaching again relaxation over longer times. The behaviour of the pore pressure $p^{(0)}_\textrm{p}$ and fluid velocity ${\varvec{w}}^{(0)}_\textrm{p}$ follows a qualitatively similar trend. For the full macroscale problem parametrisation the reader is referred to Tab. S1. To summarize, when the specimen is subject to a mechanical load, it relaxes the latter by redirecting mechanical stresses to the fluid and solid components of the tissue. The subdivision of load proportions to the vascular and interstitial compartments, together with its dynamics, is regulated by effective tensors that encode the microstructural properties of the unit cell.

In the remainder of the work we report on the influence of vascular density and tissue compressibility on the mechanical response of a tissue specimen. We consider the points in the parameter space of vascular volume ratio $\phi _\textrm{v}$ and drained Poisson ratio $\nu$ depicted in Fig. 4c and evaluate the vertical component of the homogenised solid stress $\sigma _z$ at the top point shown in Fig. 4a. We also show results concerning the spatio-temporal evolution of the pore pressure $p^{(0)}_\textrm{p}$ and pressure difference $\Delta p = p^{(0)}_\textrm{p}-p^{(0)}_\textrm{v}$ along the centreline depicted in Fig. 4a. We perform these analyses by investigating the impact of an additional physiological parameter, the vascular hydraulic permeability of the vessel walls $L_\textrm{p}$, on the tissue specimen mechanical response. We consider three values of $L_\textrm{p}$: (1) a low value corresponding to a healthy tissue; (2) a mid-range value that corresponds to a tissue that has been subject to normalisation treatments (Jain 2005; 3) a high value, for tissues with tumour-like characteristics (see Table 2).

**Table 2:** Values considered for the vascular hydraulic permeability of the vessel walls

State	Value
Healthy	$2.7\times 10^{-12}$
Normalised	$1.78\times 10^{-11}$
Tumour	$1.35\times 10^{-10}$

The values are expressed in units of ${\mathrm{m}}/(\textrm{Pa} \cdot \textrm{s})$ and are taken from Jain et al. (2007)

**Fig. 5:**
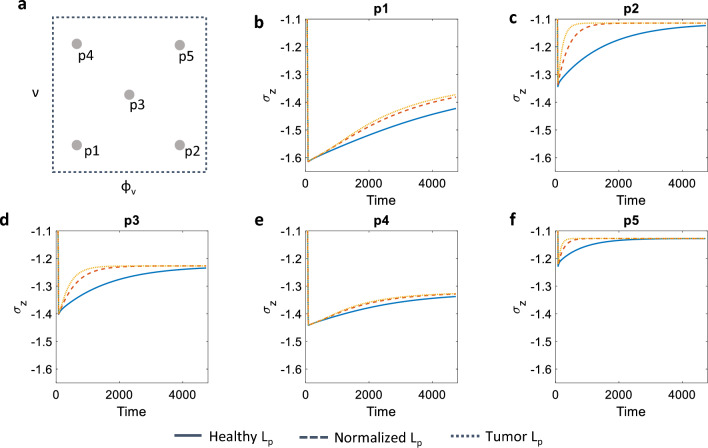
Summary of the combinations $(\phi _\textrm{v},\,\nu )$ that have been explored in the macroscale simulations (**a**). Each plot in **b**–**f** shows the temporal evolution of the vertical solid stress $(\sigma _{ \mathrm{z}})$ evaluated at the top point highlighted in Fig. 4a. Solid, dashed, and dotted lines refer to the case in which a low, mid, or high value for the vascular hydraulic permeability $L_\textrm{p}$ has been considered, corresponding to the healthy, normalized, and tumour case, respectively

Figure 5 shows the temporal evolution of $\sigma _z$, i.e. the portion of total stress carried by the solid component in a poroelastic setting, for the three conditions on the vessel hydraulic conductivity that are prescribed in Table 2. Each panel of the figure represents a position in the ($\phi _\textrm{v},\nu$) space, as specified in panel Fig. 5a. In general, the positions associated with higher vascular volume ratios are characterised by faster relaxation timescales (see Fig. 5c, f). On the other hand, low values of $\phi _\textrm{v}$ are associated with slower transients, with tissues that are not yet relaxed at the end of the simulation (as in Fig. 5b). For all the analysed conditions, higher values of $L_\textrm{p}$ correlate with faster relaxation dynamics. However, the difference in relaxation between a healthy and normalised value for $L_\textrm{p}$ is stronger than the difference between a normalised and tumour value. This might indicate a possible saturation of the dynamics at increasing values of this parameter. Finally, note that the values of $\sigma _z$ reached at the end of the simulations depend more on the value of vascular fraction $\phi _\textrm{v}$, rather than on the value of compressibility $\nu$ (compare Fig. 5b, e with Fig. 5c, f). This is once again related with the poromechanical nature of the model, and the underlying biology, in which the tight coupling between solid and fluid components in the tissue becomes evident in the partition of stresses.

**Fig. 6:**
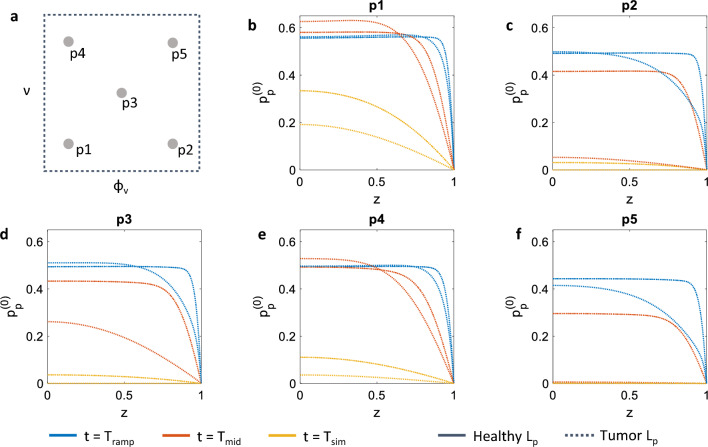
Summary of the combinations $(\phi _\textrm{v},\,\nu )$ that have been explored in the macroscale simulations (**a**). Each plot in **b**–**f** shows the spatial dependency of the fluid pressure in the poroelastic compartment $(p^{(0)}_\textrm{p})$ evaluated along the cut line highlighted in Fig. 4a. Solid and dotted lines refer to the case in which a low or high value for the vascular hydraulic permeability $L_\textrm{p}$ has been considered, corresponding to the healthy and tumour case, respectively. Different colours are used to denote the value of the variables at different times

Then, we show in Fig. 6 the evolution of the pore pressure $p^{(0)}_\textrm{p}$ for different values of vascular ratio and tissue compressibility. The pore pressure is a key regulator of fluid and solid transport in the tissue interstitial space (Jain 1994), and its role in the delivery of chemotherapies has been widely investigated (see for example Mascheroni and Schrefler 2018 and references therein). Pressure plateaux correlate to hindered delivery of therapeutic agents, whereas steep gradients are involved in fast clearance of the drug from the interstitial space. For the analysis, we considered only the extreme values of vascular hydraulic conductivity for the sake of easiness of visualisation. Again, the faster equilibration dynamics are obtained for the cases corresponding to the higher values of vascularisation, i.e. higher values of $\phi _\textrm{v}$. In both Fig. 6c, f the profile of $p^{(0)}_\textrm{p}$ at $t=T_\textrm{sim}$, the final simulation time, is fully relaxed to zero for the tumour $L_\textrm{p}$ case. This indicates fast fluid flow, with a tissue that exhibits a reduced capacity of retaining possible therapeutic molecules. The case of low compressibility and low vascularisation (p1 in Fig. 6b), on the other hand, is still in the equilibration phase at the later stages of the simulation, even for the higher value of vessel hydraulic conductivity. In all conditions, increasing $L_\textrm{p}$ leads to less steep pressure gradients at the analysed time points. This results, through Eqs. (6) and (7), to smaller fluid velocities—an indication that most of the fluid is already escaped from the specimen.

**Fig. 7:**
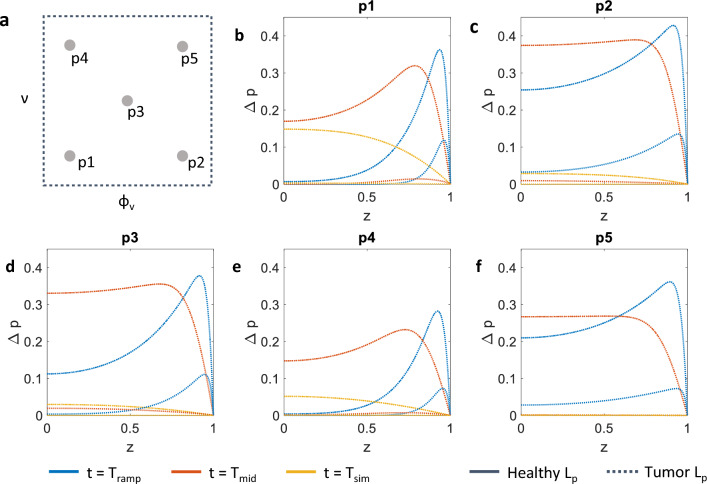
Summary of the combinations $(\phi _\textrm{v},\,\nu )$ that have been explored in the macroscale simulations (**a**). Each plot in **b**–**f** shows the spatial dependency of the difference in fluid pressure between the poroelastic and fluid compartments $(\Delta p = p^{(0)}_\textrm{p} - p^{(0)}_\textrm{v})$ evaluated along the cut line highlighted in Fig. 4a. Solid and dotted lines refer to the case in which a low or high value for the vascular hydraulic permeability $L_\textrm{p}$ has been considered, corresponding to the healthy and tumour case, respectively. Different colours are used to denote the value of the variables at different times

Finally, Fig. 7 shows the pressure difference $\Delta p$ between the poroelastic and vascular compartments at different coordinates in the vascularisation-compressibility plane. This pressure jump is related to the degree of hydraulic connectivity of the two compartments, a key regulator in drug delivery to tissues (Mascheroni and Penta 2017; Stylianopoulos et al. 2018). Indeed, low-pressure differences between the interstitial (poroelastic) and vascular compartments are associated with hindered drug transport by fluid advection, resulting in poor drug perfusion of solid tumours (Jain 1994). The simulations from the model show that, even if they reach high values of $\Delta p$ at early times, the cases corresponding to high vascularisation display the lowest pressure jumps at the end of the simulations. This is part of the transport paradox occurring in tumours: even though tumour tissues exhibit increased vascularisations and permeabilities, therapeutic molecules suffer from poor advective flows into the interstitial space. This happens because transvascular gradients are annihilated by the same factors that disregulate fluid flow across the vessel walls (Jain 1990). Decreasing compressibility (by considering higher values of $\nu$) is also associated with lower-pressure differences on the long term. Note that these outcomes are exacerbated by increasing $L_\textrm{p}$ to tumour-like values (dotted lines). These results have profound implications for the pharmacological treatment of solid tumours. Indeed, the model suggests that by modulating the vascular density (through normalization treatments) and the tumour compressibility (e.g. by employing suitable matrix remodelling agents), it would be possible to modify the dynamics of transport in the interstitial space, thus benefiting the outcome of anti-cancer therapies.

Such mechanically inspired treatments are currently the object of a vivid research in both the biological and clinical community. For example, in Tolaney et al. (2015) the authors report the results of a phase II clinical trial in which the benefits of an antiangiogenic therapy (a vessel normalization treatment) are evaluated for breast cancer patients. They conclude that the functionality of the vascular network in tumours displaying high vascular densities might benefit from “the pruning of certain vessels and increased function of the remaining, normalized vessels” (Tolaney et al. 2015). This would be qualitatively supported by the results of Fig. 7, where a reduction of vascular density correlates to higher-pressure differences between compartments.

In Chauhan et al. (2013) and Provenzano et al. (2012), the authors study the influence of extracellular matrix composition on tumour response to therapies. They find that tumour matrices are characterized by abnormal concentrations of matrix proteins, such as collagen and hyaluronan. These biopolymers contribute to the hindered transport of therapeutics in the tumour by decreasing the open interstitial space and by contributing to mechanical stresses acting to compress blood vessels (Stylianopoulos et al. 2013, 2018). The experimental work presented in Chauhan et al. (2013) and Provenzano et al. (2012) shows that by carefully delivering enzymatic agents or other therapies that normalize the abnormal tumour stroma it is possible to improve tissue perfusion and consequently to increase the action of standard chemotherapies. These findings are in qualitative agreement with the results from our mathematical model that are displayed in Fig. 7. Here, a decrease in tumour incompressibility (i.e. lower values of $\nu$) leads to higher-pressure jumps across the interstitial and vascular compartments. Nonetheless, the experimental investigators describe better perfusion as a consequence of reduced mechanical stresses compressing the vessels. In our results, increased intercompartmental pressure differences are due to a different distribution of the external loading across the matrix and fluid space of the porous material. Even if pointing to the same effect, the two explanations for improved perfusion do not thus match—even though the two explanations are not conflicting and could therefore possibly coexist. We believe the repartition of mechanical stresses in biological tissues to be an underestimated effect (appreciable only in descriptions that make use of porous media mechanics), and we advocate experiments that could shed light on this interesting phenomenon.

Finally, we refer the interested reader to the work in Stylianopoulos et al. (2018), which reviews other applications of vascular and mechanical normalization treatments to tumours. The reported experimental findings support the role of such normalization therapies in improving tissue perfusion, an insight that it is possible to draw (at least for the vascular part) also from our mathematical model. This is encouraging, as it suggests the soundness of the assumptions underlying model derivation. Nonetheless, we conclude this section by remarking that all comparisons with experiments from the biomedical literature should be treated with care and only on a qualitative level. Careful designed experiments should be carried out to test specific parts of the model under controlled conditions, in which model parameters could be suitably calibrated and experimental and theoretical results are compared quantitatively.

## Conclusions

In this work we carried out a quantitative analysis on the role of interstitial compressibility and vascular volume fraction in vascularized tissues. We performed numerical simulations of the biomechanical model presented in Penta and Merodio (2017), which describes biological tissues as porous material with double porosities (i.e. the vascular space and the interstitial space). The mathematical model is derived following asymptotic homogenization, a technique that encodes microstructural effects on the macroscopic tissue scale through the definition of effective tensorial quantities. These mathematical terms are first calculated by solving differential problems on the microscale unit cell and are then plugged into the macroscale fluid transport and mechanical balance equations.

The solution for the cell problems at the microscale showed that both vascular density and interstitial compressibility have a profound effect on the effective stiffness of the tissue, with lower compressible—lower vascularized microstructures resulting in higher effective stiffness. The microscale Young modulus, instead, shows only a multiplicative effect—as already discussed in Dehghani et al. (2018). We then simulated an experiment of stress relaxation at the tissue macroscale, in which a tissue specimen is subject to a compressive strain and the mechanical stresses and fluid pressures are recorded over time. The different fluid transport properties between the vascular and interstitial compartments indirectly allowed to prescribe a pressure jump between the vascular and poroelastic compartment, reproducing (just for a transient of time) the homeostatic conditions that allow convective transport of solutes in healthy tissues. We investigated the induced fluid dynamics for a variety of physiological parameters, involving vascular density, compressibility of the interstitial part and hydraulic conductivity of the vascular walls.

A fundamental microstructural parameter of the model is the vascular volume fraction $\phi _\textrm{v}$, quantifying the portion of unit cell that is occupied by blood vessels. We showed in the Results section that this variable has a strong influence on the mechanical response of the homogenised continuum, even though vascular density is not a direct mechanical variable by itself (compared to stiffness or shear moduli, for example). As the degree of vascularisation in a tissue might be impacted by pathophysiological conditions, especially in tumours (Carmeliet and Jain 2000), the model allows to track the influence of this variable on the final mechanical properties of the tissue, something that might be overlooked by traditional modelling approaches.

Our simulations suggest that by modulating vascular density and interstitial space compressibility it would be possible to fundamentally alter fluid transport dynamics in tissue. These modifications could be imparted on the tissue by judicious delivery of normalizing therapies and matrix remodelling agents. In particular, tissues with higher compressibilities and lower vascular densities show the highest transvascular gradients at later times (i.e. higher-pressure jumps) between the vascular and interstitial compartments. The presence of such pressure difference would allow for improved advection of drugs across the vessel walls, a condition that is known to support effective delivery of therapies to tumours (Jain 1994, 2005; Jain et al. 2007).

We recognize that the model (Penta and Merodio 2017) and its implementation herein presented rely on several simplifying assumptions and is open to improvements. One crucial limitation of the current framework is the assumption of a periodic unit cell at the microscale. This choice leads to a straightforward calculation of the effective tensors, which need to be computed only once in the whole analysis. This strong simplification could formally be relaxed by prescribing continua that are not macroscopically uniform. With this kind of assumption, only local periodicity is required and periodic unit cells are prescribed to vary parametrically with respect to the macroscale coordinate. As a result, the effective tensorial quantities become a function of the macroscale coordinate (Penta et al. 2015) and should be computed for each point of the macroscale grid.

Secondly, the model (Penta and Merodio 2017) relies on adopting a characteristic parabolic profile for the fluid vessels’ network, as previously done in Penta et al. (2014, 2015). Such an assumption leads to effective porous media governing equations at the macroscale of either Darcy’s or Biot’s type depending on whether solid deformations are considered, respectively, see, e.g. Penta and Gerisch (2017) and Penta et al. (2020), respectively. However, we are aware of other choices that had been documented in the literature, which can for instance lead to a macroscale viscoelastic behaviour, as also remarked by the authors in Burridge and Keller (1981).

Another limitation is related to the simplification that was made in including tumour vascular geometry in the model—see Fig. 1. Tumour vessels are generally abnormal (Carmeliet and Jain 2000; Baluk et al. 2005), displaying high tortuosities and poor hierarchical structure. For the sake of simplicity, we considered a simple, highly ordered geometrical configuration for the vessels in the unit cell. This issue should be addressed for a more faithful representation of the tumour tissue in the future, in order to address the interplay between the tumour poromechanics and the geometry of the vessels. In fact, vessels’ tortuosity can affect blood, drug and heat transport in solid tumours, as shown for instance in Penta and Ambrosi (2015), Mascheroni and Penta (2017), Al Sariri and Penta (2022) and Al Sariri et al. (2023). The results obtained this way could then be compared with recent permeability multiscale models such as those developed for bone tissues (Abdalrahman et al. 2015; Teo and Teoh 2012) in order to shed light onto the influence of complex fluid flow in the microvessels on the poromechanical behaviour of multiscale physical systems.

In an attempt to reduce the complexity of the traction, we embraced directly (Penta and Merodio 2017) as a starting point, so mechanical isotropy is assumed at the microscale. This constitutes a strong assumption when dealing with biological materials, especially in the case of well-organized systems as the bone or the brain (Feng et al. 2013; Scheiner et al. 2016). Mechanical anisotropy plays also an important role in multiscale models, and it can also arise based on purely geometric considerations (Penta and Gerisch 2015). However, our computational platform could be readily adapted to more complex constitutive behaviours arising for example from generalisations of Penta and Merodio (2017), as well as microscale architectures (Penta and Ambrosi 2015).

Finally, we derived the model equations in the context of the linear theory: we described the mechanical response of the matrix in terms of infinitesimal strains and adopted linear elasticity as the constitutive choice. This assumption is valid only for small perturbations imparted to the tissue specimens and is not able to capture more complex biological scenarios [e.g. growth and remodelling (Mascheroni et al. 2018)]. Nonetheless, there are developments in the recent literature (e.g. Morin et al. 2018; Miller and Penta 2021b) that address this issue in the context of multiscale models. We plan to extend the current framework to account for such large strains effects and render a more accurate description of biological phenomena.

The current analysis focuses mainly on poromechanical effects. It is known that these constitute only half of the picture, when describing phenomena that occur in biological tissues. Indeed, a part of equal importance is played by solute transport in the tissue, which is coupled (in both ways, due to osmotic effects) to poromechanical responses. A straightforward approach would be to integrate the modelling framework for solute transport presented in Penta et al. (2015) and Mascheroni and Penta (2017) into the current mathematical model. This would lead to better descriptions of biological tissues and could potentially be used to support the investigation of drug transport in pathological conditions.

Finally, it is well recognised that tissue components are mechanosensitive (De Belly et al. 2022), and mechanosensation has been identified as a modulator of pharmacological treatments (Rizzuti et al. 2020). Biological cells sense the level of mechanical stress in the environment and adapt to it by changes in their function or differentiation. At the moment, the literature of asymptotic homogenisation applied to biological tissues only describes a one-way feedback between cells and their environment: We model a unit cell (in the sense of asymptotic homogenisation) with some microstructural properties, which follow by the activities of biological cells. The microstructural properties dictate the shape of effective tensors, which regulate the macroscopic response. We propose as a natural step forward in the modelling process to “close the loop”, i.e. to take into account the influence of macroscopic stresses on the unit cell. From the biological point of view, macroscopic stresses act on biological cells, and the latter modify the tissue microscopic landscape (Mammoto et al. 2012; Bershadsky et al. 2003). This interaction could be captured phenomenologically, by modelling changes in the unit cell at the microscopic scale modulated by the stress levels at the tissue macroscale. The importance of this coupling between micro- and macroscale depends on the relative dynamics between cell mechanosensation and tissue functionality. In some pathological conditions, especially in the case of chronic inflammation or repeated injuries, this coupling between scales is of uttermost importance and its understanding could greatly benefit from mathematical models.

### Supplementary Information

Below is the link to the electronic supplementary material.

Supplementary file 1 (pdf 359 KB)

## Data Availability

Not applicable.
