# Supplementary material for: The impact of vascular volume fraction and compressibility of the interstitial matrix on vascularised poroelastic tissues
Source: Biomech Model Mechanobiol. 2023 Aug 17;22(6):1901–17. doi: 10.1007/s10237-023-01742-1 (PMC10613172; doi:10.1007/s10237-023-01742-1)
Supplement: Supplementary file 1 — (pdf 359 KB) [file 10237_2023_1742_MOESM1_ESM.pdf]

# Appendix A

| Parameter | Symbol | Value | Unit |
|---|---|---|---|
| Height of domain | H | 1 | cm |
| Radius of domain | R | 1 | cm |
| Duration of mechanical loading | $T_{ramp}$ | 10 | s |
| Time-point for observation | $T_{mid}$ | 1 | min |
| Duration of simulation | $T_{sim}$ | 10 | min |
| Imposed compressive strain | $\varepsilon_0$ | 0.05 | - |
| Fluid viscosity | $\mu$ | $4 \times 10^{-3}$ | Pa·s |
| Characteristic macroscale length | L | 1 | cm |
| Characteristic microscale length | d | 40 | $\mu$m |
| Hydraulic conductivity of poroelastic compartment | k | $2.1 \times 10^{-13}$ | $m^2/(Pa \cdot s)$ |

**Table S1** Values of the parameters that have been considered in the macroscale problem.

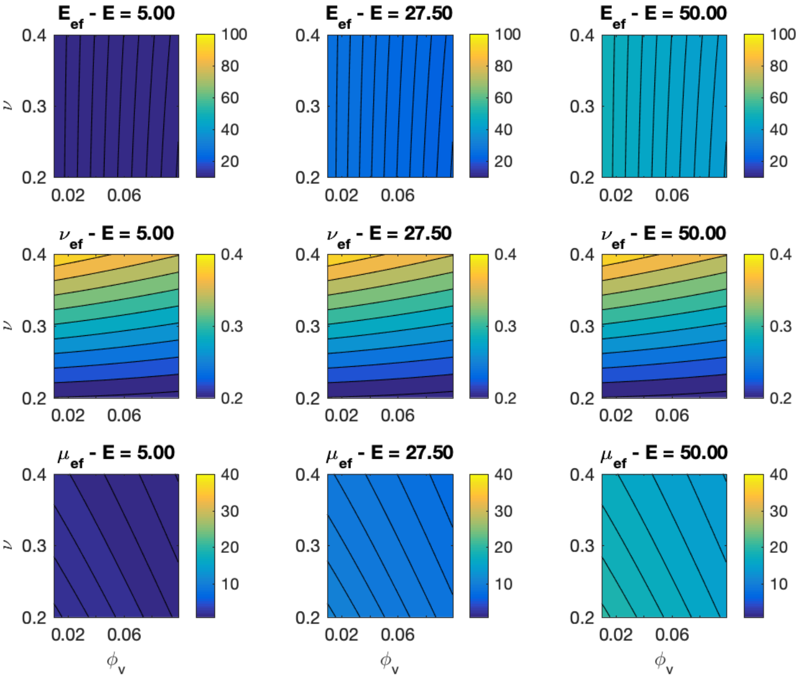


**Fig. S1** Effective Young modulus ($E_{\mathrm{ef}}$), Poisson ratio ($\nu_{\mathrm{ef}}$), and shear modulus ($\mu_{\mathrm{ef}}$) calculated from the cell problems at the microscale. In each plot, the quantity of interest is evaluated for different values of vascular fraction ($\phi_{\mathrm{v}}$) and compressibility ($\nu$). We display the results for three values of the microscale Young modulus (i.e., $E$), representing softer to stiffer tissues.

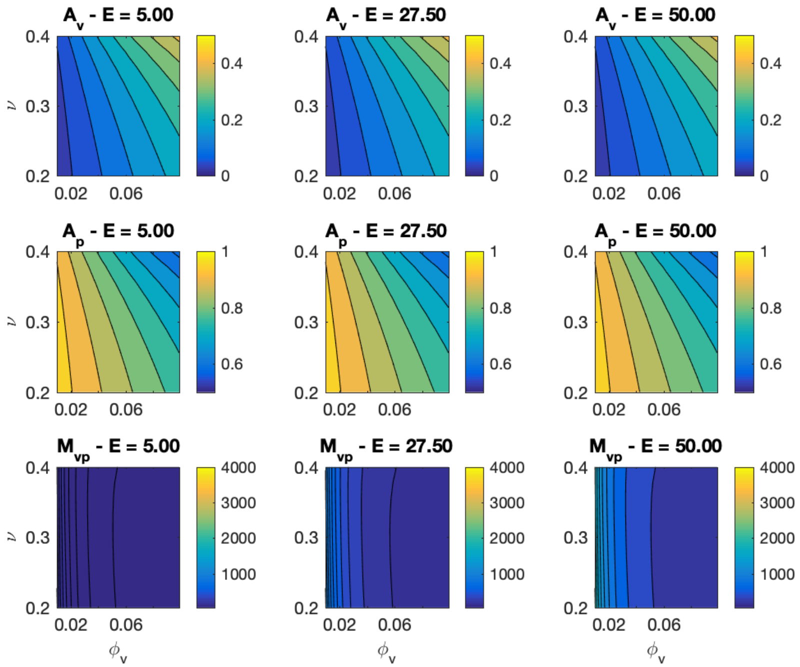


**Fig. S2** Effective vascular ($A_{\mathrm{v}}$) and poroelastic ($A_{\mathrm{p}}$) Biot coefficients, and geometric Biot modulus ($M_{\mathrm{vp}}$) calculated from the cell problems at the microscale. In each plot, the quantity of interest is evaluated for different values of vascular fraction ($\phi_{\mathrm{v}}$) and compressibility ($\nu$). We display the results for three values of the microscale Young modulus (i.e., $E$), representing softer to stiffer tissues.

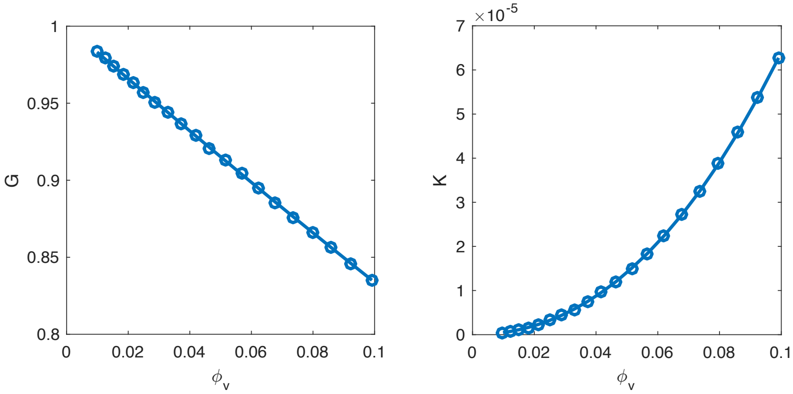


**Fig. S3** Hydraulic conductivity tensors for the vascular ($\mathsf{K}$) and poroelastic ($\mathsf{G}$) compartments, plotted as a function of the vascular volume ratio $\phi_{\mathrm{v}}$.
